# Why pathogens matter for meeting the united nations’ sustainable development goal 6 on safely managed water and sanitation

**DOI:** 10.1016/j.watres.2020.116591

**Published:** 2021-02-01

**Authors:** Alexis L. Mraz, Innocent K. Tumwebaze, Shane R. McLoughlin, Megan E. McCarthy, Matthew E. Verbyla, Nynke Hofstra, Joan B. Rose, Heather M. Murphy

**Affiliations:** a Water, Health and Applied Microbiology Lab (WHAM Lab), Philadelphia, PA USA; b Department of Public Health, College of Health and Exercise Science, The College of New Jersey, Ewing, NJ USA; c Department of Epidemiology and Biostatistics, College of Public Health, Temple University, Philadelphia, PA, USA; d Department of Civil, Construction, and Environmental Engineering, San Diego State University, San Diego, CA, USA; e Water Systems and Global Change Group, Wageningen University & Research, Wageningen, the Netherlands; f Department of Fisheries and Wildlife, Michigan State University, East Lansing, MI, USA; g Department of Pathobiology, Ontario Veterinary College, University of Guelph, Guelph, ON, Canada

## Abstract

Water and wastewater utilities, water and sanitation hygiene (WASH) practitioners, and regulating bodies, particularly in developing nations, rely heavily on indicator microorganisms, as opposed to pathogens, for much of their regulatory decisions. This commentary illustrates the importance of considering pathogens and not relying only on indicator organisms when making decisions regarding water and sanitation, especially with respect to meeting the current targets of the Sustainable Development Goal (SDG) 6. We use quantitative microbial risk assessment (QMRA) to present three common scenarios that WASH and public health practitioners encounter to illustrate our point. These include 1) chlorination of surface water for drinking, 2) land application of latrine waste as a fertilizer, and 3) recreation/domestic use of surface waters impacted by wastewater discharge. We show that the calculated probabilities of risk of infection are statistically significantly higher when using treatment/survival information for pathogens versus using indicator species data. Thus, demonstrating that relying solely on indicators for sanitation decision making is inadequate if we truly want to achieve the SDG6 targets of safely managed water and sanitation services.

## Highlights

• Indicator organisms *do not* tell the whole story for the safety of water and sanitation systems.
• Considering *only* fecal indicator groups may provide a falsely reduced sense of risk.
• Consideration of *pathogens matters* for meeting SDG6.

## Graphical abstract

**Figure:**
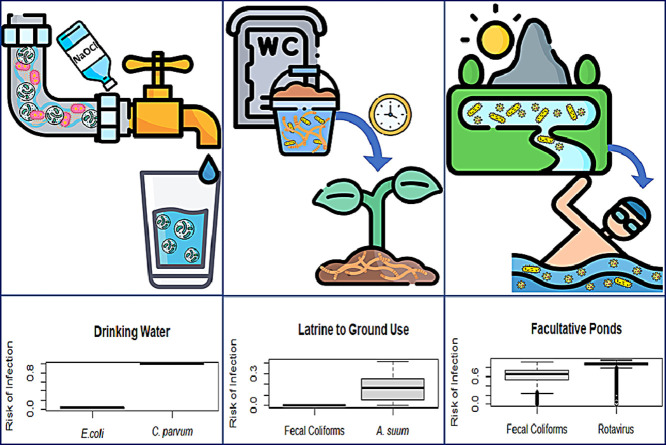

## 1 The status quo in water and sanitation

As the global water and sanitation community moves from a goal of “improved” drinking water sources and sanitation systems to “safely managed”, as per the United Nations Sustainable Development Goal 6 (SDG 6), the conversation inevitably needs to include a discussion on pathogens. Unsafe sanitation and/or the unsafe management and discharge of excreta to the environment leads to surface and groundwater contamination and human exposure to pathogens (Mitchell et al., 2016; Peal et al., 2014; WHO, 2006). The World Health Organization (WHO) recently released guidelines on sanitation and health that aim to provide water and sanitation and hygiene (WASH) practitioners with information on pathogens to guide investments and interventions in order to improve overall waterborne disease-related health outcomes (UN, 2018; World Health Organization, 2018). However, the guidelines need to be more widely promoted among water and wastewater utilities, WASH practitioners and regulating bodies in low income countries who rely largely on indicator microorganisms and often have limited knowledge on pathogens (Bain et al., 2014; Figueras and Borrego, 2010; Saxena et al., 2014). For example, in a study conducted in Uganda, among water and sanitation practitioners, only a few stakeholders reported being very familiar with pathogens, and less than half correctly identified fecal coliforms as being bacteria (Tumwebaze et al., 2019). From a compliance-monitoring standpoint, the use of indicator microorganisms such as fecal (thermotolerant) coliforms, *E. coli* (*E. coli*) or fecal *enterococci,* is a practical approach. Indicators are easier and less costly to analyze than pathogens (Edberg et al., 2000; Pype et al., 2016). Indicators are also more abundant in the environment than most pathogens, so culture-based methods allow for easy enumeration (Espinosa et al., 2009). Their presence can indicate contamination with human or animal feces (McGinnis et al., 2018). Thus, these organisms are often used as a measure of treatment performance in the sanitation chain (Bain et al., 2014; Gerba, 2009; Silverman et al., 2013). This use is misleading since indicators belong predominantly to the bacteria group and they are often less persistent and more easily inactivated during treatment processes, compared to many viruses, protozoa or helminth eggs (Arthurson, 2008; Savichtcheva and Okabe, 2006).

In many countries, sanitation efforts are scaling up and WASH practitioners, city planners and engineers are designing and implementing sanitation interventions. However, studies show a significant amount of pathogen-contaminated fecal waste continues to reach the environment due to inadequate containment and treatment (WHO/UNICEF, 2019; WWAP, 2018). Thus, with the current SDG 6 targets of achieving safely managed water and sanitation services, it is important that WASH practitioners advocate for, and implement appropriate technological, treatment and management systems for the removal of pathogens (Ingallinella et al., 2002; Katukiza et al., 2010; Vagadia, 2018; Wu et al., 2016).

Although higher income countries are further along in terms of achieving “safely managed” sanitation, the discussion is also relevant there. Rural populations across North America and Europe rely on septic systems for on-site sanitation. Septic systems are not designed for pathogen reduction and setback distances between septic systems and private wells are often inadequate for protecting against sewage entering private water supplies (Murphy et al., 2020; Schaider et al., 2016). In addition, many cities in Europe and North America utilize combined sewer systems where raw sewage is discharged during rain events into urban waterways (EPA, 2016). Combined sewer overflows contribute to the 90 million illnesses in the US that are estimated to be attributed to recreational waterborne diseases annually (DeFlorio-Barker et al., 2018; McLellan et al., 2018).

The objective of this commentary is to illustrate the importance of considering pathogens and not relying only on indicators when making decisions regarding water and sanitation to meet the SDG 6′s targets on safely managed drinking water and sanitation services. Specifically, when evaluating the performance of treatment systems or survival of pathogens in excreta, it is critical that we do not rely solely on indicator data when making decisions. The goal of this commentary is not to advocate for routine monitoring of pathogens, rather to ensure that they are considered in the design of treatment systems and interventions, particularly in low income contexts that lack data on pathogens and rely heavily on indicator microorganism data when making decisions on water and sanitation safety. The Global Water Pathogen Project (GWPP) and the World Health Organization (WHO) have resources (WHO n.d) and tools (www.waterpathogens.org/tools) (K2P Tools) available that can support sanitation decision-making while considering the importance of pathogen reduction along the sanitation service chain.

We present three common scenarios that WASH and public health practitioners encounter in low income countries to illustrate our point on how relying on indicator microorganisms alone for making decisions related to the treatment of water or excreta can underestimate the true health risks for exposure to pathogens.

1. Chlorination of unfiltered surface water for drinking water and risks to consumers (Hunter, 2009).
2. Land application of latrine waste as a fertilizer and risks to farmers (Dey et al., 2016).
3. Recreational/domestic use of surface waters impacted by wastewater discharge and risks to recreators (Tilley, 2014).

We use quantitative microbial risk assessment (QMRA) to demonstrate the difference in infection risk when one relies only on indicator data instead of pathogen-specific information. The scenarios presented below are intended to show differences in infection risk, and not to be used as average or global risks of infection for each scenario. The scenarios presented are purposefully broad and simplified to illustrate this point. It is important to note that each of these scenarios focuses on only one pathogen and the overall risk of *all* infections would be higher. QMRA is a quantitative approach to assessing public health risk by estimating risk of infection and illness when a population is exposed to a pathogen in the environment (Haas et al., 2014).

## 2 The importance of pathogens

Guidelines on sanitation and data on the survival of pathogens in sanitation systems have evolved with the help of freely accessible, evidence-based information, such as that provided through the Global Water Pathogen Project (GWPP) database (Rose and Jiménez-Cisneros, 2017). The GWPP is an open-access online database and knowledge platform, providing information on water-related disease risk and intervention measures (waterpathogens.org). The database is designed to enable ongoing updates via a network of experts to provide up-to-date information on new and emerging pathogens (Rose and Jiménez-Cisneros, 2017).

Pathogens belong to four broad groups (Rose and Jiménez-Cisneros, 2017):

• viruses – the smallest of all the infectious organisms consisting of genetic material (DNA or RNA) enclosed within a protein capsid, some of which are encased in an envelope; require a host cell to replicate (size 0.02–0.75 µm)
• bacteria – small, single-celled organisms, many of which are capable of multiplication outside a host given favorable conditions (size 1–5 µm)
• protozoa – complex and relatively large, single-celled organisms, some (enteric protozoa) cannot replicate outside a suitable host (size 10–55 µm)
• helminths – complex multi-cellular organisms, often designated as “worms”; their life-cycles may involve other host animals; largely transmitted through the fecal route with various exposure pathways (size 15–35 cm)

Water and wastewater utilities have traditionally used indicator organisms to serve as surrogate organisms to point out the presence of certain pathogens in water and sanitation systems (Fuhrimann et al., 2015; Howard et al., 2006; Nabateesa et al., 2017). However, some studies have shown that the measurements of single indicator organisms do not correlate with pathogens (Harwood et al., 2005; Wu et al., 2011). Wu et al. (2011) assembled a dataset containing 540 cases from studies that investigated relationships between pathogens and indicators. After assessing the pathogen-indicator relationships, it was found that only 223 (41.3%) of them were correlated. Similarly, Harwood et al. (2005) found that no single indicator organism correlated with the pathogens studied in reclaimed water, suggesting that additional monitoring of pathogens is fundamental to protect public health.

Among other factors, these studies presumably reflect the differences in persistence and survivability of pathogenic vs. non-pathogenic microorganisms. For example, in a study of the persistence of enterotoxigenic *E. coli* (ETEC), Lothigius et al. (2010) found that the species had a 4 log_10_ reduction after 69.5 days at 21 °C under natural sunlight conditions. Conversely, under similar light and temperature (25 °C) conditions, Dick et al. (2010) found that non-pathogenic (indicator) *E. coli* had a 4 log_10_ reduction after only 2.18 days (Dick et al., 2010; Lothigius et al., 2010). The failure of many studies to demonstrate a relationship between indicators and pathogens is in part the result of these differences in persistence.

## 3 Indictors miss the mark when predicting health risks

**Fig. 1:**
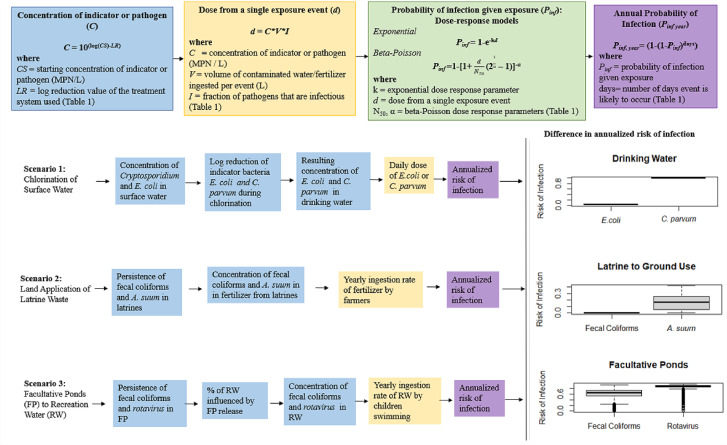
QMRA Framework, Exposure Scenarios and Comparison of Risk from Indicator Species and Pathogens Row 1-The generalized risk framework is represented in top row with each step color coded to make these steps clear in each scenario-specific row (blue for the concentration calculations, yellow for the dose by exposure calculation, green for the probability of infection calculations, and purple for the annualization of the risk of infection). Row 2-The second row represents Scenario 1: In this scenario, surface water is chlorinated for drinking water purposes without the use of filtration. It shows the risk of *C. parvum* infection when using the survival information for an indicator species, *E. coli*, versus the pathogen, *C. parvum* following chlorine disinfection*.* Row 3*-* The third row represents Scenario 2: In this scenario latrine waste from a lime treated pit latrine is used as fertilizer in land application. It shows the risk of *A. suum* infection when using the survival information for an indicator species, fecal coliforms, versus the pathogen, *A. suum* in the lime treated pit latrine*.* Row 4*-* The fourth row represents Scenario 3: In this scenario a facultative pond discharges into surface water that children recreate in. It shows the risk of a rotavirus infection when using the survival information for an indicator species, fecal coliforms, versus the pathogen, rotavirus through the facultative pond. For each scenario, the annualized risks of infection for the indicator species and the pathogen are represented using boxplots.

Three QMRA models were developed to illustrate how applying indictor treatment/survivability data as a proxy for pathogen reduction can significantly underestimate potential health risks (Fig. 1). The QMRAs in this paper were developed in the statistical programming language R version 3.5.3, using treatment, survival and concentration data on pathogens and indicators available in the literature (R Development Core Team, 2008). Annual risk of infection was chosen as the end point for the models. Risk estimates were generated using Monte Carlo simulation (10,000 iterations). A schematic of the risk framework is presented in Fig. 1. The QMRA approach is described in detail in Haas, Rose, and Gerba (Haas and Rose, 2014). Data used in the models were sourced from the literature and are presented in Table 1. Indicator species and pathogens for each exposure scenario were chosen based on: 1) the indicator species being commonly used in risk assessments for the exposure scenario 2) the pathogen being a common cause of disease due to the exposure scenario, and 3) availability of robust survival and treatment data specific to the exposure scenario.

**Table 1:** Concentration, persistence, dose, and dose-response values used in the QMRA model.

*Table 1 represents the parameters used in the QMRA model. These parameters were obtained from the literature, as cited. The concentration of the indicator species and pathogens used in each example were taken from studies with similar conditions. The ingestion parameters were obtained from observational studies under the conditions described in each scenario (daily drinking water consumption, farmers ingesting excreta, and children recreating in water). The dose response parameters represent the dose-response curve for infection after the pathogen has been ingested. The confidence intervals and standard deviations from the data sources are noted as needed. The distributions were modelled through monte-carlo simulations in the QMRA model. The following notations are used in the table: *k*=growth rate, ID50=infectious dose for 50% of the population, SD=standard deviation, CI=95% confidence interval, α= beta distribution parameter, N_50_ = dose at which 50% of the population is affected, FF = fluorescent foci (unit for rotavirus).

See Refs.Kahn and Stralka, 2009; Messner et al., 2001; Navarro et al., 2009; Ward et al., 1986

Table 1 presents the starting concentrations for the pathogens in each scenario, the log reductions for pathogens and corresponding indicator reductions in each scenario, the dose response models selected along with other relevant assumptions. Paired pathogen/indicator survival and treatment data from the literature was used for all three scenarios. The only difference in the QMRA models presented under each scenario between the indicator species and the pathogen is the treatment/survival data. We use this approach to show the difference in calculated risk when using indicator species survival data as a proxy for pathogen survival data. The QMRA results by scenario are presented as boxplots in Fig. 1.

### 3.1 Scenario 1: chlorination of drinking water

Chlorination is a commonly used water treatment option globally. Although coagulation, flocculation, and sedimentation/filtration are often recommended prior to disinfection in surface water, in developing countries, surface water is often chlorinated for drinking water at the point of use (Levy et al., 2014). In scenario 1, surface water used for drinking is treated with chlorine. The annualized risk of a cryptosporidiosis infection is calculated using survival/ treatment data for the pathogen *Cryptosporidium parvum* (*C. parvum*) and the common indicator bacteria *E. coli* when treated with free chlorine (Douglas, 2015). *Cryptosporidium* was selected as the pathogen in this scenario as it is very resistant to chlorine (Korich et al., 1990). The authors would like to note that the concentration of *C. parvum*, used in this scenario, 26.3 oocytes/L is quite high. *C. parvum* can have a wide range, but this specific point estimate comes from a surface water study conducted in Thailand which the authors consider representative of surface water quality found in many low-income countries (Anceno et al., 2007).

*E. coli* was selected as the indictor organism as it is frequently used as a measure of drinking water quality in low income settings (WHO, 2017). The corresponding survival data for these organisms under chlorination are presented in Table 1. Data on typical drinking water consumption from a study in the United States was used as consumption data in developing countries is lacking. A mean 1233 mL/ day (CI: 1200–1265 mL/ day) was used to calculate daily exposure to *Cryptosporidium* (Table 1).

In this scenario, the median annualized probability of infection with cryptosporidiosis when assessed using the persistence values for *E. coli* during free chlorine treatment is 0.03 as compared to 0.99 when using the persistence values for *C. parvum* (Douglas, 2015). This means that when assessing the risk of cryptosporidiosis using survival values for *E. coli* during free chlorine treatment, an individual has approximately a 3% chance of developing the infection in a year, versus a 99% chance of developing the infection when using the persistence data for *C. parvum.* When comparing the difference in risk using a *t*-test, the *p*-value is <0.0001. This further illustrates that although *E. coli* is effectively reduced by chlorination, *C. parvum* is practically unaffected by chlorine and therefore using *E. coli* as a proxy for treatment performance may be misleading and significantly underestimate the potential health risk (Korich et al., 1990).

This difference in median annualized risk of infection is supported by the Harwood et al. (2005) study of six wastewater reclamation facilities. The authors found no significant correlations between any indicator organism (e.g., fecal coliforms and F-specific coliphages) and any pathogenic organism (e.g., enteric viruses, *C. parvum*, and *Giardia*). For example, fecal coliforms were found in 27% of the disinfected effluent samples, while *C. parvum* oocysts were found in 70% of the same samples (Harwood et al., 2005). When the data for all indicators were used for discriminant analysis, the authors were only able to predict the presence/absence of pathogenic organisms. Accordingly, this suggests that the use of a suite of indicators and the implementation of consistent pathogen monitoring is better suited to protect public health than using a single indicator organism (Harwood et al., 2005).

### 3.2 Scenario 2: latrine waste applied to land as a fertilizer

Fecal sludge has a history of land application, such as being used as a fertilizer (Jayathilake et al., 2019). Since it is rich in vital nutrients, fecal sludge is also applied to agricultural lands to replenish those nutrients which have been depleted from the soil. This practice has important applications for resource recovery, as 22% of the total global phosphorus demand could be supplied by the available phosphorus in human excreta (Mihelcic et al., 2011). While many governments and governmental organizations regulate its application, others do not (Jayathilake et al., 2019). For example, the US EPA requires that septage is treated and stabilized if it will be applied directly to land. It must undergo aerobic digestion, anaerobic digestion, air drying, composting or co-composting, and/or lime stabilization (Jayathilake et al., 2019). Untreated fecal sludge can also be applied to land if its attractiveness to insects and rodents is diminished through injection, immediate incorporation or lime stabilization (Jayathilake et al., 2019). However, neither set of regulations specifies quality parameters such as the concentrations of indicators and/or pathogens within the fecal sludge. Therefore, the safety of the soil for agricultural use is largely assumed based on expected treatment efficiencies/decay rates. Farmers, particularly in low income settings, who contact such soils without adequate protective gear and/or using improper handling methods, may risk being infected with a variety of pathogens, for example, helminths such as *Ascaris* spp. (Dey et al., 2016).

In Scenario 2, waste from a lime-treated latrine is used as fertilizer and the risk of ascariasis in farmers is being compared when using the persistence data of fecal coliforms, a common bacterial indicator, to that of the pathogen *Ascaris suum* (Endale et al., 2012). The data used for the starting concentrations of *Ascaris suum* in the lime treated pit latrine and the corresponding persistence data for *Ascaris suum* and fecal coliforms in the pit latrine all come from one study from Ethiopia (Endale et al., 2012). The exposure to excreta for farmers was taken from a study from Vietnam that estimated involuntary ingestion of excreta (Van Vu et al., 2018; Table 1). The resulting median annual probability of infection with *Ascaris suum* when assessed using the persistence values for fecal coliforms during lime latrine treatment is 0.00 as compared to 0.11 when using the persistence values for *Ascaris suum.* It is important to note, the risk of disease is never 0, implying there is no risk, but rather, in this case the risk is incalculably small. This means, when assessing the risk of ascariasis infection using fecal coliform persistence data, we would not expect an individual to become infected in a year, whereas if we use the *Ascaris suum.* persistence data, an individual has approximately an 11% chance of being infected in a year. When comparing the difference in risk using a *t*-test the *p*-value is <0.0001. The risk of ascariasis infection when determined using *A. suum* persistence data is roughly 2-fold higher than the acceptable per person per year risk of ascariasis infection of 1.2 × 10^−3^ (or 10^−5^ Disability Adjusted Life Years (DALY) loss per person year (Mara and Sleigh, 2010). This is based off of the tolerable waterborne disease threshold of less than or equal to 10^−6^ DALY loss per person year (WHO 2008). This risk of infection to risk of disease translation is based on ingesting *A. suum* eggs while eating raw vegetables (Mara and Sleigh, 2010). This example highlights the fact that helminth eggs are generally much more resistant to chemical treatment (such as lime) compared to bacteria such as *E. coli*.

### 3.3 Scenario 3: recreational use of surface water contaminated with wastewater discharge

Inadequately treated wastewater and/or untreated wastewater threaten the quality of surface waters which are used for recreational activities such as swimming or religious activities. Communities situated downstream or near municipal sewage outfalls are at the highest risk of illness due to microbial contamination from polluted effluents (Naidoo and Olaniran, 2013). Specifically, water bodies used for full contact recreation activities, or bathing (such as in low income countries) can serve as a source of infection via ingestion or through full body contact (Fuhrimann et al., 2016a; Mitch et al., 2010; Okoh et al., 2010; Rose and Jiménez-Cisneros, 2017). A study conducted in Uganda reported an increased risk to gastrointestinal pathogens among populations that were exposed to untreated wastewater in Kampala open drains and waterways (Fuhrimann et al., 2016a).

In Scenario 3, the risk of a rotavirus infection in children recreating in surface waters after discharge from a facultative holding pond (commonly used treatment in low income settings) is calculated using the persistence data of fecal coliforms and the pathogen, rotavirus, in a facultative stabilization pond (Table 1). Data on survival of viral pathogens in facultative ponds (also known as waste stabilization ponds) are lacking, the data in risk models developed herein were taken from a study that documented the persistence of rotavirus in an experimental pond with a hydraulic retention time of 5–6 days (Oragui et al., 1995; Pearson et al., 1995). Corresponding survival data for fecal coliforms in a facultative pond with the same retention time (5–6 days) were used (Von Sperling, 1999). The exposure to the pond effluent in the receiving surface water body was calculated by first estimating the dilution factor (Sukias et al., 2001) and by estimating the amount ingested during swimming which was taken from a study conducted by Dufour et al., 2017. Dufour and colleagues documented volumes of water ingested during swimming by testing for cyanuric acid excreted in the subjects’ urine (Dufour et al., 2017). The number of swimming days per year was taken from two studies in Kampala, Uganda (Fuhrimann et al., 2016b; Katukiza et al., 2014) .The median annual probability of infection of rotavirus when assessed using the persistence values for fecal coliforms during facultative pond treatment is 0.65 as compared to 0.89 when using the persistence values for rotavirus*.* Certain fecal coliforms, such as *E. coli,* can grow in surface waters, influencing the persistence data, and therefore the risk of infection using these data can be more conservative. When comparing the difference in risk using a *t*-test the *p*-value is <0.0001. These results depict only the risk of infection in this scenario, and not the risk of illness. This scenario is not intended to depict the average risk of rotavirus infection to children recreating in surface waters influenced by facultative ponds, but rather to show how the risk changes, using this particular example, if risk is assessed based on an indicator species rather than a pathogen.

Heightened median annualized risk of illness when using viral persistence data rather than indicator data may be explained by studies like that undertaken by Carducci et al. (2009). The team studied the influent and effluent of wastewater treatment plants to quantify reductions in concentrations of viral particles (Carducci et al., 2009). They also evaluated whether traditionally used bacterial indicators correlated to viral concentrations. The authors found that treated wastewater still contained unacceptable levels of infectious human viruses. Furthermore, they found no correlation between bacterial indicators (e.g., *E. coli* and *Enterococci*) and the viruses considered (e.g., rotavirus, norovirus I and II). Virus removal in wastewater treatment ponds is highly variable and not very efficient compared to the removal of fecal indicator bacteria (Verbyla and Mihelcic, 2015). Similarly, a South African study by Adefisoye et al. (2016) of two wastewater treatment plants found no correlation between fecal coliforms and the occurrence of human adenovirus (HAdV) in effluents (Adefisoye et al., 2016). They also found a heightened persistence of HAdV in effluents, suggesting that public health is not adequately protected by measuring only indicator organisms like fecal coliforms.

## 4 Conclusions

In this commentary we have illustrated the importance of considering pathogens and not only relying on indicators when making decisions regarding water and sanitation, which is critical as we move forward with “safely managed water and sanitation” for SDG6. In summary:

• The calculated probabilities of risk of infection are statistically significantly higher when using treatment/persistence information for pathogens versus using persistence data for indicator species in each scenario.
• Considering *only* fecal indicator groups when assessing treatment efficiencies of sanitation and drinking water treatment systems may provide a scenario with a falsely reduced sense of risk.
• Process indicators, treatment indicators, or indicators of mobility and fate are used to assess treatment or disinfection efficacy, or surface and subsurface microbial transport and should include representative organisms from the four key pathogen groups when appropriate (Farnleitner and Blanch, 2017).
• Pathogen presence and persistence are important to understand in the water and sanitation sector in order to develop more realistic interventions to avert the risk of disease to the public, sanitation workers, and WASH practitioners.
• New tools and resources that consider pathogens are available to support sanitation decision making through the WHO and the GWPP.
• Indicator species *do not* tell the whole story for the safety of sanitation systems, which is why *pathogens matter*.

## Declaration of Competing Interest

The authors declare that they have no known competing financial interests or personal relationships that could have appeared to influence the work reported in this paper.
